# Synthesis and characterization of chromium aluminum carbide MAX phases (Cr_x_AlC_x-1_) for potential biomedical applications

**DOI:** 10.3389/fchem.2024.1413253

**Published:** 2024-07-03

**Authors:** Muhammad Shahbaz, Nadeem Sabir, Nasir Amin, Zobia Zulfiqar, Muhammad Zahid

**Affiliations:** ^1^ Department of Physics, Govt College University Faisalabad, Faisalabad, Pakistan; ^2^ Punjab Institute of Nuclear Medicine (PINUM), Faisalabad, Pakistan; ^3^ Department of Chemistry, University of Agriculture Faisalabad, Faisalabad, Pakistan

**Keywords:** sol-gel, metal carbide, MAX phase, biomedical applications, *Candida albicans*, HepG2

## Abstract

MAX phases, characterized as nanolaminates of ternary carbides/nitrides structure, possess a unique combination of ceramic and metallic properties, rendering them pivotal in materials research. In this study, chromium aluminum carbide ternary compounds, Cr_2_AlC (211), Cr_3_AlC_2_ (312), and Cr_4_AlC_3_ (413) were successfully synthesized with high purity using a facile and cost-effective sol-gel method. Structural, morphological, and chemical characterization of the synthesized phases was conducted to understand the effects of composition changes and explore potential applications. Comprehensive characterization techniques including XRD for crystalline structure elucidations, SEM for morphological analysis, EDX for chemical composition, Raman spectroscopy for elucidation of vibrational modes, XPS to analyze elemental composition and surface chemistry, and FTIR spectroscopy to ensure the functional groups analysis, were performed. X-ray diffraction analysis indicated the high purity of the synthesized Cr_2_AlC phase as well as other ternary compounds Cr_3_AlC_2_ and Cr_4_AlC_3_, suggesting its suitability as a precursor for MXenes production. Additionally, the antimicrobial activity against *Candida albicans* and biocompatibility assessments against *Escherichia coli* (*E. coli*), *Staphylococcus aureus* (*S. aureus*), and HepG2 cell line were investigated. The results demonstrated significant antifungal activity of the synthesized phases against *Candida albicans* and negligible impact on the viability of *E. coli* and *S. aureus*. Interestingly, lower concentrations of Cr_2_AlC MAX phase induced cytotoxicity in HepG2 cells by triggering intercellular oxidative stress, while Cr_3_AlC_2_ and Cr_4_AlC_3_ exhibited lower cytotoxicity compared to Cr_2_AlC, highlighting their potential in biomedical applications.

## Graphical Abstract

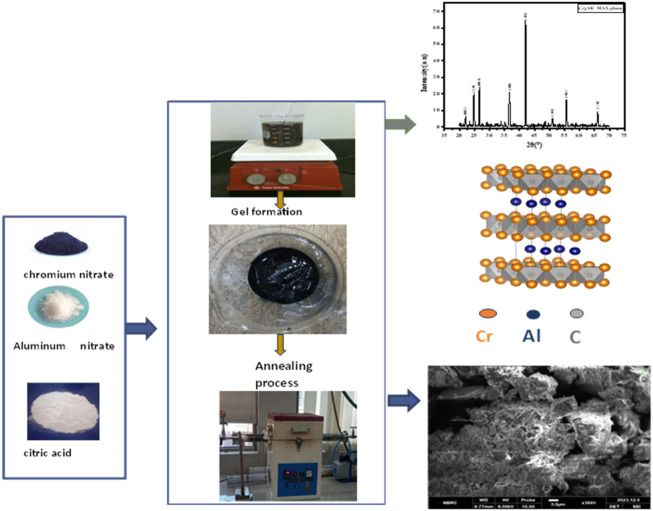

## 1 Introduction

MAX phases are synthesized by various methods at high temperatures. Different methods also prepared the Cr_2_AlC MAX phase. Here, the question is what are the lowest temperature and cost-effective achievable methods for synthesizing MAX Phases? Firstly, the Cr_2_GaC MAX phase was synthesized by the sol-gel method at low temperatures (Siebert et al., 2019). Classifications of MAX phases can be delineated according to distinct ‘n’ values, encompassing M_2_AX (211), M_3_AX_2_ (312), and M_4_AX_3_ (413) phases, respectively (Sun, 2011). The distinctive combination of weakened M-A bonds and robust M-X bonds, coupled with a nano-layered structure, imparts to these solids a unique amalgamation of metallic properties and biological applications. Other MAX phases include 312 and 413 of Ti_2_AlC (Rampai and Tokoloho, 2011; Galvin et al., 2018; Gao et al., 2020; Poulou et al., 2021). The synthesis of the Cr_2_AlC MAX phase involves various methods such as the molten salt method (Abdelkader, 2016), ball milling (Ta et al., 2021; Mansouri et al., 2023), chemical vapor deposition (CVD) (Gorshkov et al., 2017), Physical vapor deposition (PVD) (Rueß et al., 2021; Li et al., 2024) and, notably, the sol-gel method. In the previous literature, the sol-gel method was utilized to fabricate the Cr_2_GaC MAX phase (Siebert et al., 2019). This research investigated two materials, Cr_2_AlC MAX phase, and Cr_2_CT_x_ MXene-Cr, for their potential biomedical applications. Both materials showed free radical scavenging activity, with MXene-Cr being more effective. MXene-Cr also inhibited the enzyme alpha-amylase and displayed strong DNA nuclease activity. Furthermore, both materials exhibited significant antimicrobial activity against various bacteria, with better effects on Gram-positive bacteria. Notably, they inhibited microbial growth at low concentrations and MXene-Cr showed promising antibiofilm activity. Additionally, MXene-Cr demonstrated impressive antibiofilm activity against *S. aureus* (89.86%) and *P. aeruginosa* (87.01%), while the MAX phase displayed an antibiofilm activity exceeding 90%. These findings suggest promising potential for both materials in various biomedical applications (Kaya et al., 2024).

Originating from the mid-1800s, early investigations into silica gels form the foundational basis for the widely employed sol-gel chemistry, a method extensively applied in the synthesis of inorganic solids (Hench and West, 1990). Within the sol-gel methodology, the initial step entails the creation of a colloidal suspension termed “sol,” which then undergoes a structural transformation into a network referred to as a “gel.”

Various synthesis methods are utilized for Cr_2_AlC MAX phase fabrication, including the molten salt method (Liu et al., 2020; Shamsipoor et al., 2021; Liu et al., 2023), ball milling (Mansouri et al., 2023), chemical vapor deposition (CVD) (Lei and Lin, 2022), physical vapor deposition (PVD) (Gonzalez-Julian, 2021), and spark plasma sintering (Duan et al., 2015). Recent research has highlighted the potential of MAX phases as effective high-temperature coatings, with applications spanning turbojets, aircraft, automobiles, and the petrochemical industry. J Liu et al. Successfully produced Cr_2_AlC thin films, a type of MAX coating, using magnetron sputtering techniques (Li et al., 2018; Liu et al., 2018). Both elemental and compound targets are employed for the deposition process, with substrate heating or post-annealing necessary to achieve crystallization. However, despite these efforts, challenges persist in achieving the desired stoichiometric ratio and homogeneity due to the presence of binary carbides or intermetallic impurities in the resulting thin films. Manoun et al. synthesized Cr_2_AlC compounds by employing a Hot Isostatic Pressing (HIP) method. The synthesis involved mixing Cr, Al, and C, followed by subjecting the mixture to conditions of 1,200°C and approximately 100 MPa pressure (Manoun et al., 2006). Yukhvid et al. employed a self-propagating high-temperature synthesis (SHS) technique to prepare Cr_2_AlC. The synthesis involved utilizing mixtures of Cr_2_O_3_, CrO_3_, Al, and C, conducted under an argon gas atmosphere at 5 MPa pressure (Gorshkov et al., 2018). Additionally, the reaction kinetics and mechanical properties of the Cr_2_AlC compound was investigated, which was synthesized via the hot pressing (HP) technique at 1,300 °C and 30 MPa (Yan et al., 2019). Despite their merits, these methods typically necessitate elevated temperatures (>1,000°C), high pressures (up to 100 MPa), and sophisticated equipment, thereby limiting their widespread applicability (Lin et al., 2022). Previous studies have predominantly utilized various compounds such as Al_4_C_3_, AlCr_2_, Cr_2_O_3_, and CrCx (where x = 0.5) as raw materials to mitigate the risk of secondary compound formation during processing (Rajkumar et al., 2017). Generally, synthesizing the Cr_2_AlC MAX phase directly from a mixture of elemental C, Al, and Cr powders is challenging due to the likely formation of intermediate phases such as CrxCy and Al-C compounds, as well as the oxidation tendency of Al and Cr (Tian et al., 2008). Consequently, there have been limited reports on the fundamental synthesis of the Cr_2_AlC MAX phase from elemental powders without the need for high-pressure or complex tools (Yan et al., 2017; TAN et al., 2019).

The above-mentioned methods are more expensive and are high-temperature and high-pressure based. Based on the aforementioned studies, ensuring phase purity is of paramount importance for MAX phase materials. This study aimed to create a facile method for producing the Cr_2_AlC MAX phase, utilizing elemental powders as initial ingredients without applying pressure. In the initial stages, the sol-gel method was employed to prepare the Cr_2_AlC MAX phase. Additionally, the ternary compounds Cr_3_AlC_2_ and Cr_4_AlC_3_, which exhibit similarities to Cr_2_AlC MAX phase, were also synthesized using the sol-gel method by adjusting the concentration ratios of chromium nitrate for the 312 and 413 phases. Sol-gel method is cost cost-effective method and easy to maintain. It is low low-temperature-based technique for synthesizing the Cr_2_AlC MAX phase. Other phases Cr_3_AlC_2_ and Cr_4_AlC_3_, (312 and 413) are not mentioned in previous studies, but we prepared those phases successfully by using the sol-gel method. The prepared phases are more stable. we called it ternary compounds. Cr_3_AlC_2_ and Cr_4_AlC_3_, are probably MAX phases. Other novel phases of Ti_2_AlC are mentioned in the literature (Tzenov and Barsoum, 2000; Sarkar et al., 2024). The MAX phases are synthesized by the novel sol-gel route and applied for the potential biomedical applications. This work’s novelty lies in utilizing a cost-effective the sol-gel technique for synthesizing MAX phases with three different ratio. The development of novel MAX phases such as Cr_3_AlC_2_ and Cr_4_AlC_3_ was realized using the sol-gel method. The developed materials were successfully confirmed by several analytical techniques with excellent biomedical application. Herein, the facile sol-gel method has been opted for the synthesis of the Cr_2_AlC MAX phase and related ternary compounds. In the sol-gel method, nitrate precursors and citric acid are used for the synthesizing of Cr_2_AlC, Cr_3_AlC_2_, and Cr_4_AlC_3._ Comprehensive characterization through X-ray diffraction (XRD), X-ray photoelectron spectroscopy (XPS), scanning electron microscopy (SEM) with energy-dispersive X-ray spectroscopy (EDX), Fourier-transform infrared spectroscopy (FTIR), Raman spectroscopy, confirmed the Cr_2_AlC MAX phase and other prepared Cr_3_AlC_2_ and Cr_4_AlC_3_ were found to have comparable structural morphology with Cr_2_AlC MAX phase with the prepared Cr_2_AlC showing promising results across all analyses, and the ternary compounds exhibiting significant characteristics comparable to the Cr_2_AlC MAX phase, further validated through a comparative analysis with the 211 MAX phase. The prepared materials were assessed for biomedical applications including Anti-bacterial, Anti-fungal, and Anti cancerous. The anti-bacterial activity was done against *E coli* and *S. aureus,*anti-fungal activity against *Candida* albacians, and anti-cancerous activity against the HepG2 cell line (liver cancer cell). The thorough characterization and biomedical assessments underscore the potential of these materials for various applications, offering insights into their structural, chemical, and functional properties, and positioning them as valuable candidates for future research and technological advancements. The realm of material science has been perennially enriched by the discovery and synthesis of novel materials that offer a blend of desirable properties for a wide range of applications.

## 2 Materials

The chemicals used in this study are Chromium (III) Nitrate Nonahydrate [Cr(NO_3_)_3_·9H_2_O, 99.9%, Honeywell], Aluminum Nitrate Nonahydrate [Al(NO_3_)_3_·9H_2_O, 99.9%, ChemPUR], and Citric Acid [99.5%, Alfa Aesar]. The deionized water was used throughout the research. All the chemicals used were of analytical grade and used without further purification (Siebert et al., 2019).

### 2.1 Synthesis of Cr_2_AlC, Cr_3_AlC_2_, and Cr_4_AlC_3_ MAX phases

**FIGURE 1:**
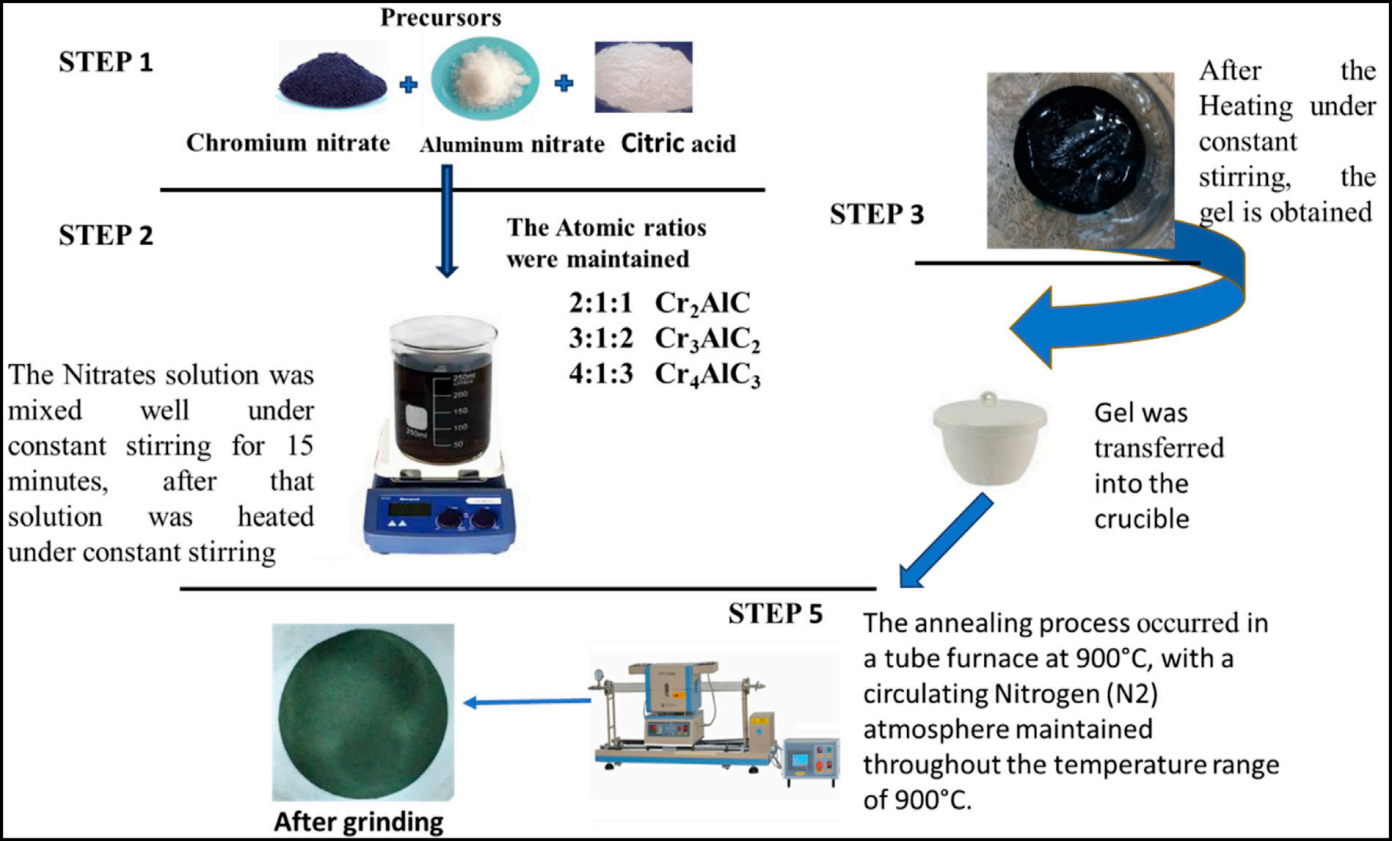
Schematic Diagram for the synthesis of Chromium Aluminum Carbide MAX Phases (Cr_x_AlC_x-1_).

In this experimental procedure, For the 2:1 ratio, 16.0 g of Cr(NO_3_)_3_·9H_2_O and 7.5 g of Al(NO_3_)_3_·9H_2_O were used as precursors, along with 8.4 g (9 equivalents) of citric acid. This same amount of Al(NO_3_)_3_·9H_2_O and citric acid was used for the 3:1 and 4:1 ratios, while the amount of Cr(NO_3_)_3_·9H_2_O was increased to 24.0 g and 32.0 g, respectively. The precursor chemicals were dissolved in water using a magnetic stirrer bar in a beaker. The resulting mixture was then heated within a temperature range of 70°C–80 °C, forming a viscous liquid. Once it gelled, the material was moved to an Al_2_O_3_ crucible for subsequent heat treatment. This annealing process occurred in a horizontal tube furnace (Carbolite) at 900 °C in a Nitrogen atmosphere (N_2_,99.99% purity), with 2 °C/min heating rate. The temperature was maintained for 5 hours before allowing the sample to cool gradually to room temperature. Various materials were heated at a temperature of 900 °C for 5 hours to gain a deeper understanding of the reaction dynamics. Following thermal treatment, the specimens underwent a 30-min grinding process. After this initial grinding, further grinding was carried out before subjecting the samples to comprehensive characterization using various analytical techniques (Siebert et al., 2019). The schematic diagram of synthesis is shown in Figure 1.

### 2.2 Characterizations

The Cr_2_AlC, Cr_3_AlC_2_, and Cr_4_AlC_3_ samples generated in the synthesis were subjected to a series of analytical techniques for comprehensive characterization. A Bruker D2 Phaser X-ray Diffractometer equipped with Cu-Kα radiation (λ = 1.5406 Å). The scanning range extended from 20° to 70° in terms of 2θ. Particle size and morphology were examined through SEM Cube II, Emcraft South Korea with EDX. Vibrational spectra were obtained using the Confocal Micro Raman MNSTEX PRI 100, DONGWOO South Korea system. The diverse stretching and bending vibrations of functional groups within the synthesized materials were identified using Fourier Transform Infrared Spectroscopy (FTIR) with an Agilent Technology Cary 360 FTIR spectrophotometer. Additionally, X-ray Photoelectron Spectroscopy (XPS) analysis, performed with ESCALAB-250 (Thermo Scientific, United Kingdom), delved into the electronic states present in the material. 1mL Dimethyl sulfoxide (DMSO) was used to prepare the 40 mg/mL dose to perform biomedical activity. Strains of *E. Coli*, B. cereus, and strains of Fungi were assembled from the Department of Microbiology lab in Govt. College University Faisalabad, Pakistan.

### 2.3 Antifungal activity

The procedure involves creating *Candida albicans* inoculum in Sabouraud Dextrose Broth, adjusting turbidity, and applying MAX Phase materials to agar plates (Bakht et al., 2011). Plates were incubated, and inhibition zones around the MAX Phase material were measured to assess antifungal effectiveness.

### 2.4 Anti-bacterial activity

This involves preparing *E. coli* inoculum in LB broth, followed by turbidity adjustment, and applying MAX Phase materials on agar plates. Followed by incubation, zones of inhibition are observed to assess antibacterial effectiveness the same procedure applied for Cr_3_AlC_2_ and Cr_4_AlC_3_.

### 2.5 Anti-bacterial activity against *S. aureus*

A standardized procedure was opted by preparing *S. aureus* inoculum, applying MAX Phase materials on Mueller-Hinton agar plates, and observing inhibition zones after incubation to assess antibacterial effectiveness (Sharmin et al., 2021). The same method was applied for Cr_3_AlC_2_ and Cr_4_AlC_3_.

### 2.6 Anti-cancerous activity

Protocol for assessing cytotoxicity on HepG2 ([HEPG2]-HB-8065-ATCC) cells includes culturing cells, treating with Cr_2_AlC MAX phase materials, performing MTT assay, and analyzing data to determine IC50 value (Younas et al., 2021). The systematic approach ensures accurate evaluation of cytotoxic effects.

## 3 Results and discussion

### 3.1 Material’s characterization

**FIGURE 2:**
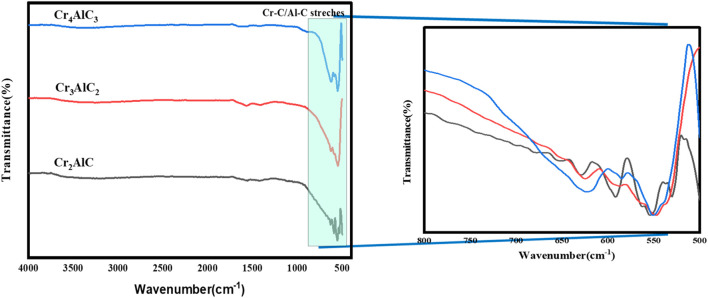
FTIR Analysis of prepared samples of Cr_2_AlC, Cr_3_AlC_2_, and Cr_4_AlC_3_.

The analysis using Fourier transform infrared (FTIR) spectroscopy was performed to ascertain the chemical properties of the synthesized material. FTIR analysis was accomplished to illustrate the surface functional groups present in the Cr_2_AlC, Cr_3_AlC_2_, and Cr_4_AlC_3_ (Figure 2). The peaks observed between 430 and 820 cm^−1^ corresponded to the stretching vibration modes of Al-O and Cr-O bonds, indicating the presence of Cr-C and Al-C bonds (Reghunath et al., 2021) for Cr_2_AlC, Cr_3_AlC_2_, and Cr_4_AlC_3_. The FTIR characteristics of prepared Cr_3_AlC_2_ and Cr_4_AlC_3_ were aligned with the FTIR spectrum of the Cr_2_AlC MAX phase. Peaks appeared between 550 and 800 cm^-1^ are ascribed to the Al-C bonding. In all the FTIR spectra the presence of peaks can be realized confirming the Al-C stretching vibration mode (Shalini Reghunath et al., 2021).

**FIGURE 3:**
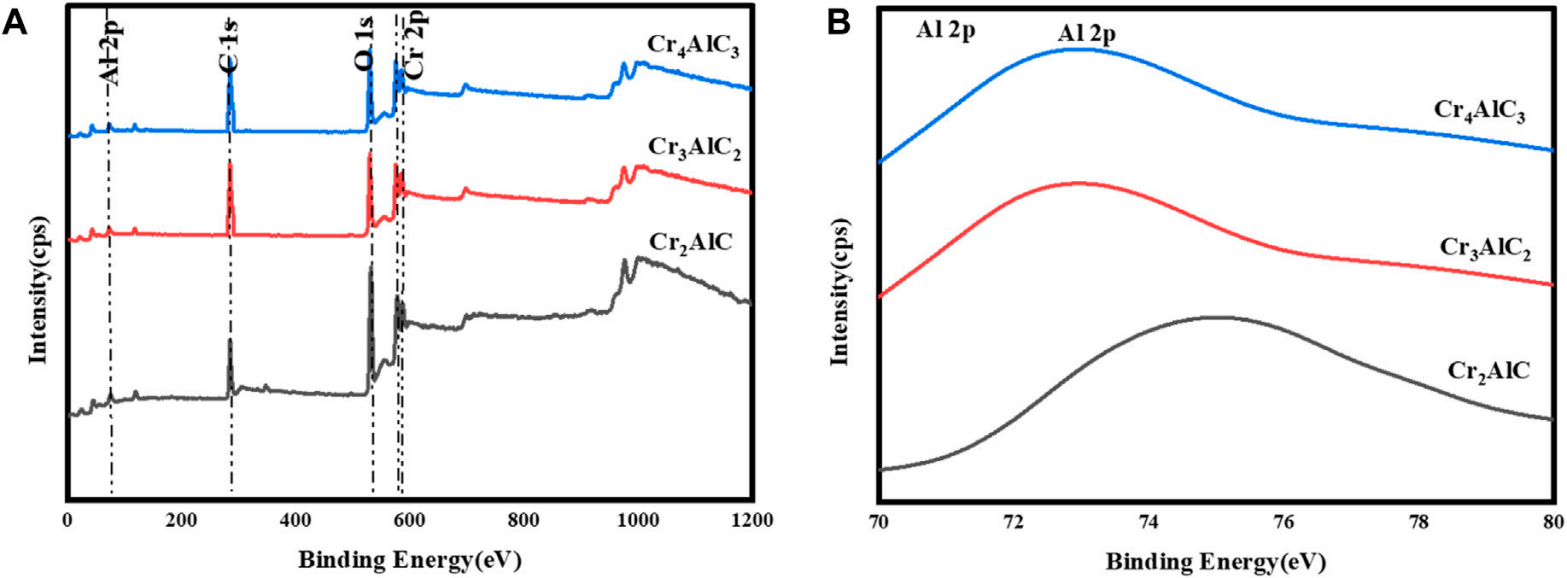
**(A)** XPS analysis of prepared Cr_2_AlC, Cr_3_AlC_2_, and Cr_4_AlC_3,_ **(B)** High resolution XPS spectra of Al 2p.

**FIGURE 4:**
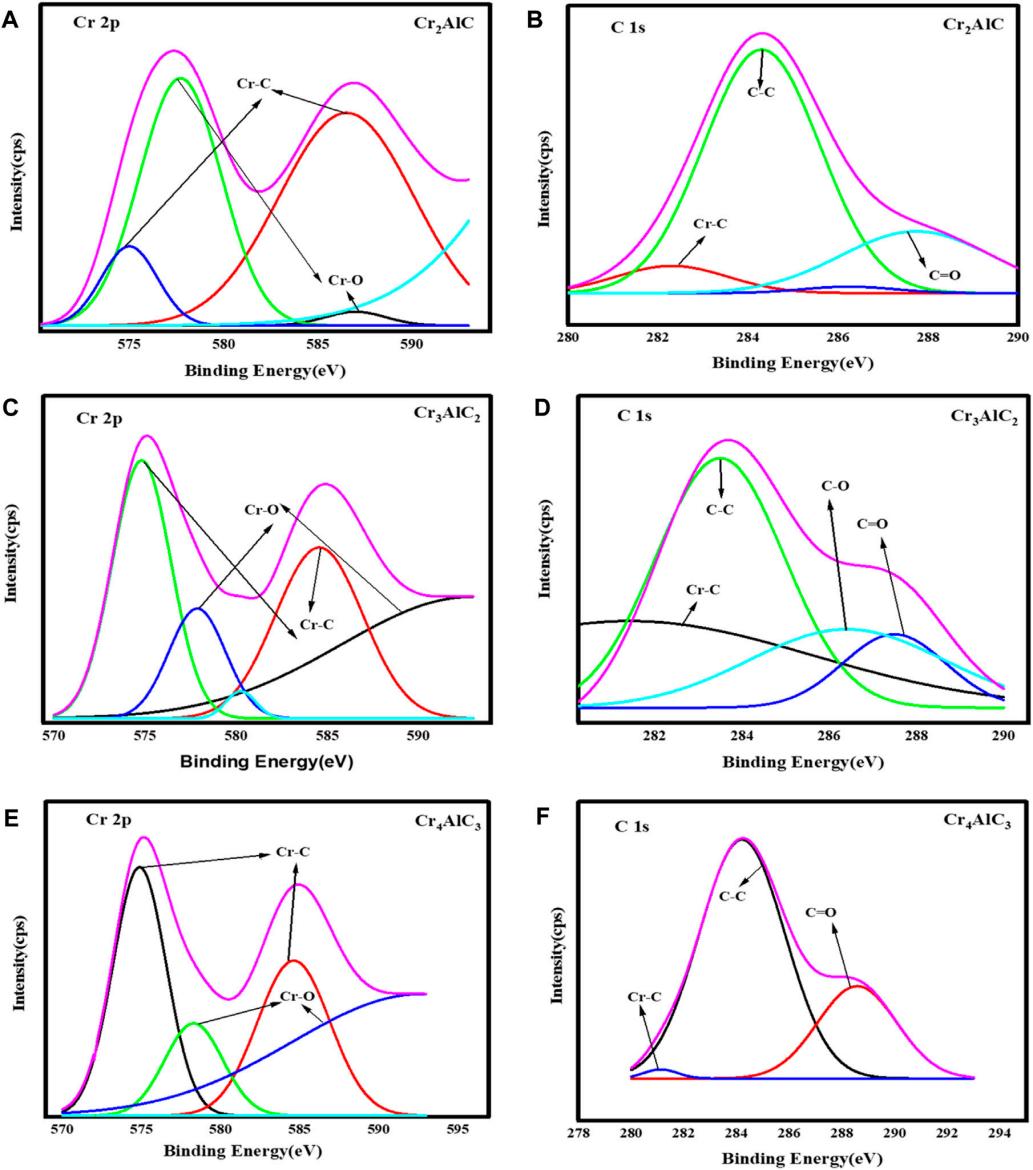
High resolution XPS spectra of **(A, B)** Cr 2p&C 1s for Cr_2_AlC, **(C,D)** Cr 2p&C 1s for Cr_3_AlC_2_, and **(E,F)** Cr 2p&C 1s for Cr_4_AlC_3_.

To investigate the surface composition and valence states of the Cr_2_AlC MAX phase, XPS analysis of its structural elements was conducted. The results are illustrated in Figure 3. The survey scan of the Cr_2_AlC Max phase, Cr_3_AlC_2_, and Cr_4_AlC_3_ are presented in Figure 3A. The presence of Cr, Al, O, and C elements was confirmed, aligning with findings from prior research (Wei et al., 2015; Soundiraraju et al., 2020). In the high-resolution spectra of the Cr_2_AlC Max phase, the Cr spectrum (Figure 4A) exhibits discernible peaks at 575.0 eV and 586.4 eV for Cr 2p (i.e., 2p3/2 and 2p1/2 respectively. These peaks are indicative of the presence of the Cr–C bond characteristic of chromium carbide, consistent with findings reported by Zamulaeva and co-workers (Zamulaeva et al., 2016). Peaks corresponding to Al 2p were detected at 73.9 eV (Figure 3B) (Hauert et al., 1993), attributed to the Al-C bond. The strong reactivity between aluminum and carbon facilitates the formation of a layered Cr_2_AlC structure, characterized by alternating layers of chromium and aluminum. In the C1s spectrum (Figure 4B), a peak at 282.4 eV is attributed to the Cr-C bond (Zamulaeva et al., 2013). Therefore, the high-resolution XPS results suggest the formation of a high-purity Cr_2_AlC MAX phase (Monireh et al., 2023a). In Cr_3_AlC_2_, the Cr spectrum (Figure 4C) exhibits discernible peaks at 574.8 eV and 584.6 eV for Cr 2p (i.e., 2p3/2 and 2p1/2 respectively. These peaks are indicative of the presence of the Cr–C bond characteristic of chromium carbide. Peaks corresponding to Al 2p were detected at 73.1 eV (Figure 3B), attributed to the Al-C bond. In the C1s spectrum (Figure 4D), a peak at 282.6 eV is attributed to the Cr-C bond. In Cr_4_AlC_3_, the Cr spectrum (Figure 4E) exhibits discernible peaks at 574.8 eV and 584.6 eV for Cr 2p (i.e., 2p3/2 and 2p1/2 respectively. These peaks are indicative of the presence of the Cr–C bond characteristic of chromium carbide. Peaks corresponding to Al 2p were detected at 73.0 eV (Figure 3B), attributed to the Al-C bond. In the C1s spectrum (Figure 4F), a peak at 281.1 eV is attributed to the Cr-C bond. All the results of XPS for Cr_3_AlC_2_ and Cr_4_AlC_3_ aligned with the Cr_2_AlC MAX phase. There are some shifts in peaks, which may be due to material and may be due to changes in composition.

**FIGURE 5:**
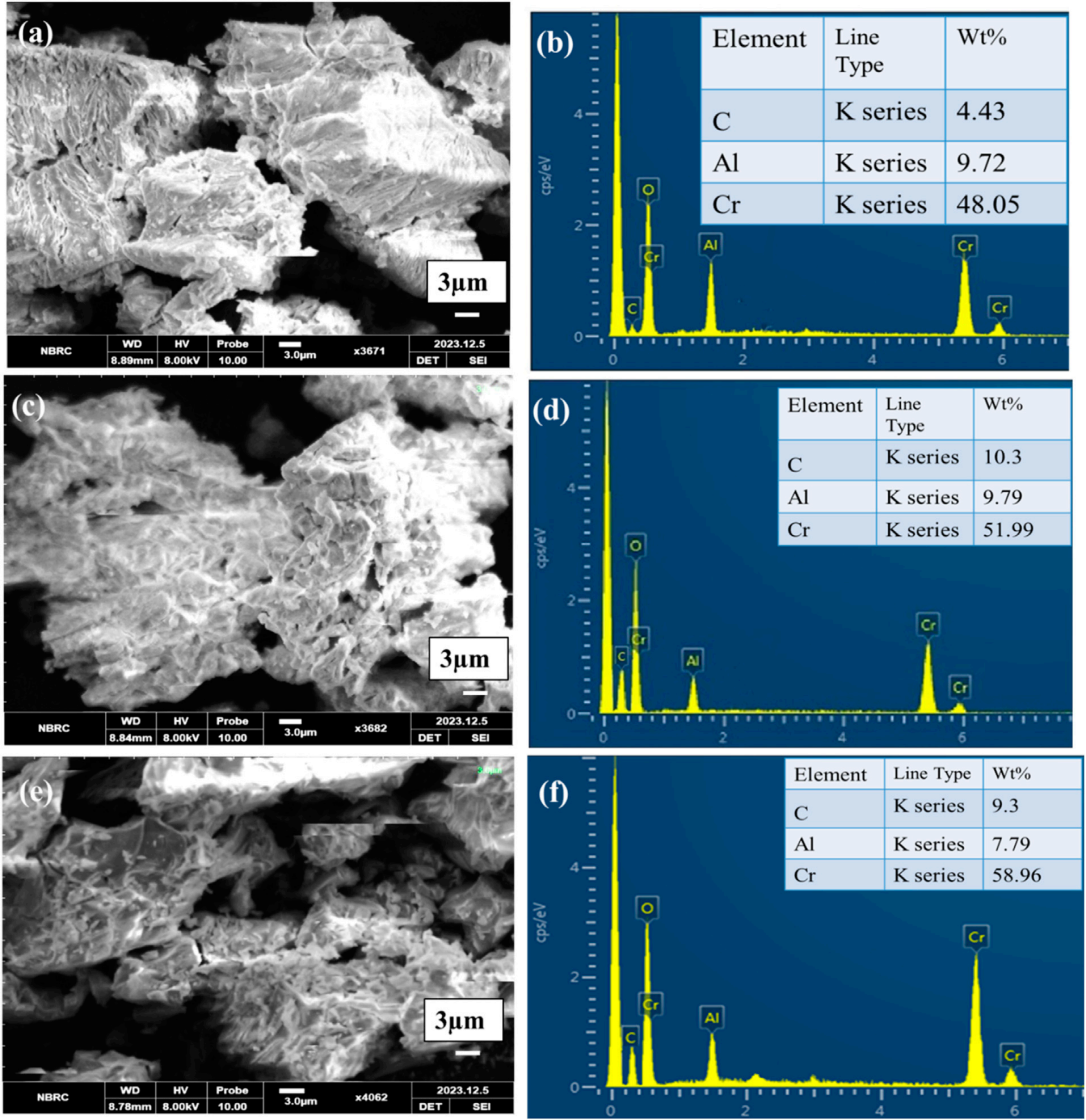
SEM-EDX analysis **(A,B)** Cr_2_AlC, **(C,D)** Cr_3_AlC_2_, and **(E,F)** Cr_4_AlC_3_.

The morphologies of prepared Cr_2_AlC, Cr_3_AlC_2_, and Cr_4_AlC_3_ were examined by Scanning electron microscope (SEM). The lamellar sheet structure, characteristic of the prepared materials is evident in Figures 5A, C, E. The layers exhibit delamination, with noticeable kink bands present. The uneven structural morphology with varying surface energies reflects versatility in structural composition thereby effective physicochemical characteristics of the materials. All the results of Cr_3_AlC_2_, and Cr_4_AlC_3_ agreed with Cr_2_AlC. The lamellar sheets were nonuniform in thickness (Sharma and Pandey, 2019; Sharma et al., 2023).

The elemental analysis of the Cr_2_AlC, Cr_3_AlC_2_, and Cr_4_AlC_3_ powder (Figures 5B, D, F) verified the presence of carbon (C), chromium (Cr), aluminum (Al), and oxygen (O), with no detection of additional elements. Atomic percentages within the EDX spectrum are reported, and the observed values closely correspond to the formula of Cr_2_AlC, Cr_3_AlC_2_, and Cr_4_AlC_3_ (Monireh et al., 2023b). The expected stoichiometric ratios for Cr_2_AlC, Cr_3_AlC_2_, and Cr_4_AlC_3_ are 2.5:1:1.0, 2.8:1:2.3 and 3.9:1:2.7 for Cr:Al: C respectively. The obtained ratios closely match this, with a slight excess of Chromium (Cr). These ratios indicate a composition of approximately Cr_2.5_Al_1_C_1.0_, Cr_2.8_Al_1_C_2.3_, and Cr_3.9_Al_1_C_2.7_ suggesting a nearly stoichiometric Cr_2_AlC, Cr_3_AlC_2_, and Cr_4_AlC_3_ with a slight deviation. This deviation could be due to experimental error, sample inhomogeneity, or actual variations in stoichiometry. The purity of the prepared material in EDX is evident as it only contains Cr, Al, C, and O. There are no other elements present.

**FIGURE 6:**
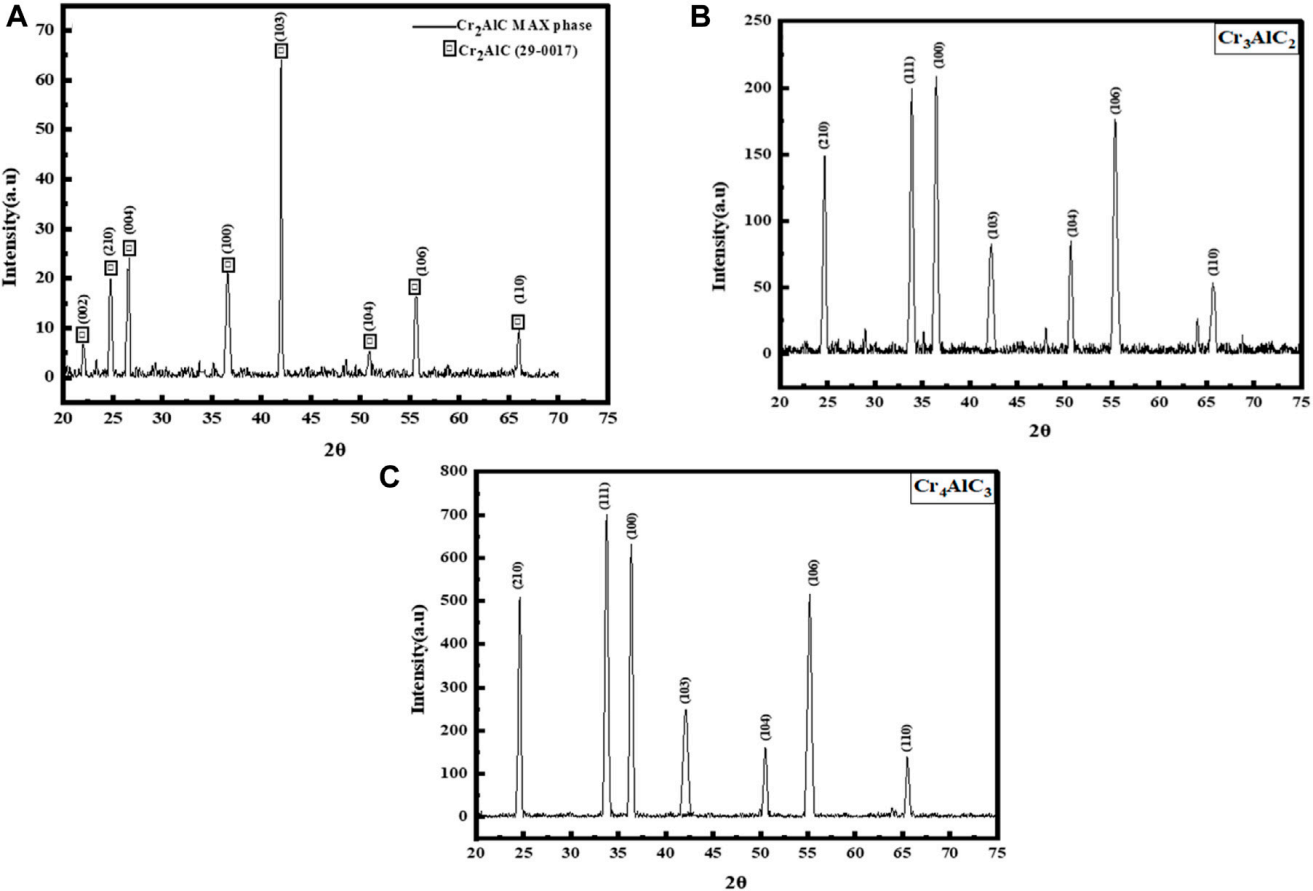
XRD analysis of prepared samples of **(A)** Cr_2_AlC, **(B)** Cr_3_AlC_2_, and **(C)** Cr_4_AlC_3_.

The XRD pattern of the Cr_2_AlC MAX phase as prepared is displayed in Figure 6A. The distinct sharp peaks observed confirm the formation of the Cr_2_AlC MAX phase. Furthermore, the discernible peaks observed at the 2θ = 22.0^°^, 24.7^°^, 26.5^°^, 36.6^°^, 42.1^°^, 50.9^°^, 55.6^°^ and 65.9^°^ correspond to the (0 0 2), (2 1 0), (0 0 4), (1 0 0), (1 0 3), (1 0 4), (1 1 6) and (1 1 0) planes of the synthesized sample, respectively. This observation affirms the hexagonal structure of the prepared MAX phase (Crisan and Crisan, 2018; TUNES et al., 2021). For Cr_3_AlC_2_ and Cr_4_AlC_3_ (Figures 6B, C, respecctively) all the 2θ values corresponding to the planes were matched with the Cr_2_AlC MAX phase. This finding validates the hexagonal structure of the prepared Cr_3_AlC_2_ and Cr_4_AlC_3_. Furthermore, the crystallite size of the Cr_2_AlC was determined to be 55.4 nm using the Debye–Scherrer equation (Zhang et al., 2019) based on the intense XRD peak observed at 2θ = 42.1 (Guan, 2016). Furthermore, the Crystallite size of the Cr_3_AlC_2_ and Cr_4_AlC_3_ were determined to be 23.1 nm and 21.3 nm using the Debye–Scherrer equation, based on the intense XRD peak observed at 2θ = 33.70^°^and 33.50^°^ respectively and the Crystallite size Cr_3_AlC_2_ and Cr_4_AlC_3_ were also determined to be 21.8 nm and 23.6 nm using the Debye–Scherrer equation, based on the intense XRD peak observed at 2θ = 36.4^°^ and 36.39^°^respectively. The values of the lattice constant (a and c) for the hexagonal pattern were designed employing Eq. 1. The equation represents the inter-planar spacing (dhkl) for the plane (hkl) and is expressed as:

$$\frac{1}{d^{2}}=\left(\frac{h^{2}}{a^{2}}+\frac{hk}{a^{2}}+\frac{k^{2}}{a^{2}}+\frac{l^{2}}{c^{2}}\right)$$ (1)

The determined unit cell parameters are a = 3.03 Å and c = 12.3 Å for the Cr_2_AlC MAX phase (Siebert et al., 2019; Reghunath et al., 2021). The unit cell parameters have been determined as follows: for the Cr_3_AlC_2_, a = 2.46 Å and c = 10.28 Å, and for the Cr_4_AlC_3_ plane, a = 2.47 Å and c = 10.31 Å. The X-ray diffraction (XRD) analysis confirmed the hexagonal structure of the synthesized material. This is further supported by the match between the observed peaks and the reference pattern in JCPDS card no. 29–0017 (Shalini Reghunath et al., 2021). The a and c parameters for Cr_3_AlC_2_, and Cr_4_AlC_3_ aligned with the Cr_2_AlC. Cr_3_AlC_2_ and Cr_4_AlC_3_ can be probably called MAX phases.

**FIGURE 7:**
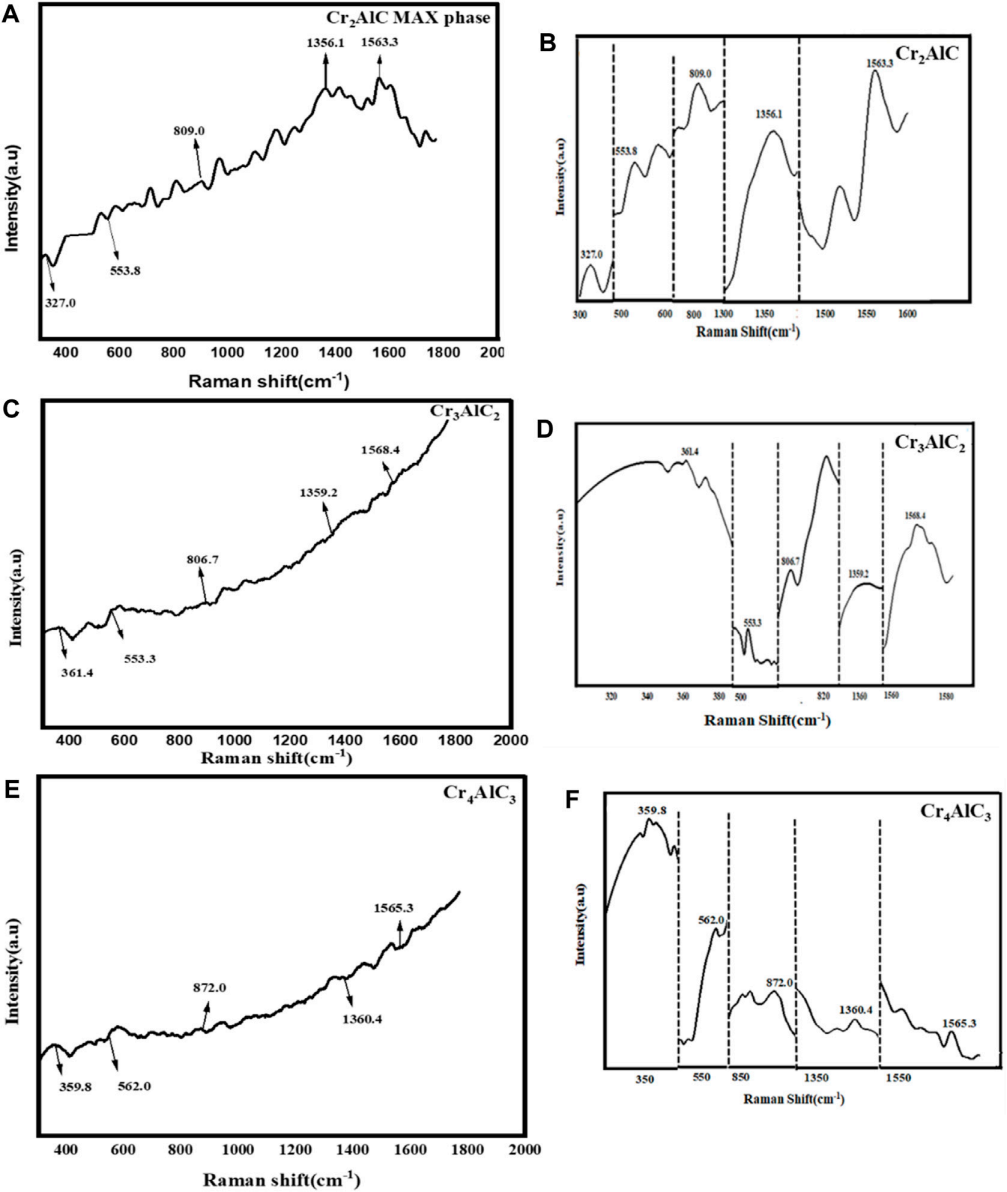
Raman spectra for **(A,B)** Cr_2_AlC, **(C,D)** Cr_3_AlC_2_, and **(E,F)** Cr_4_AlC_3_.

Raman spectroscopy was performed on the samples across a spectral range from 400 cm⁻^1^–2000 cm⁻^1^, as illustrated in Figure 7. The observed peaks at 327 cm⁻^1^ and 553.8 cm⁻^1^,361.4 cm⁻^1^ and 553.3 cm⁻^1^ correspond to the signature peaks of CAC(Cr_2_AlC) and Cr_2_O_3_, confirming the formation of Cr_2_O_3_ due to surface oxidation. The obtained results have been confirmed form several relevant publications (Spanier et al., 2005; Shtansky et al., 2009; Bortolozo et al., 2010; Presser et al., 2012; Vishnyakov et al., 2014; Bentzel et al., 2016; Zhang et al., 2021). Additionally, the peak observed at 809.0 cm⁻^1^, 806.7 cm⁻^1^ and 872.0 cm⁻^1^ in the Raman spectra of Cr_2_AlC, Cr_3_AlC_2_, and Cr_4_AlC_3_ respectively is attributed to the vibrational mode of the carbide (Bortolozo et al., 2007). The Raman spectrum showed the D band at a Raman shift of 1,356.1 cm⁻^1^,1359.2 cm⁻^1^ and 1,360.4 cm⁻^1^ and the G band at a Raman shift of 1,563.3 cm⁻^1^,1568.4 cm⁻^1^, and 1,565.3 cm⁻^1^ for Cr_2_AlC, Cr_3_AlC_2_, and Cr_4_AlC_3_ respectively. Carbon’s D peaks are observable at 1,356.1 cm⁻^1^,1359.2 cm⁻^1^ and 1,360.4 cm⁻^1^ and G peaks at 1,563.3 cm⁻^1^,1568.4 cm⁻^1^, and 1,565.3 cm⁻^1^ for Cr_2_AlC, Cr_3_AlC_2_, and Cr_4_AlC_3_ respectively. The G band is referred to as the graphitic band. Its presence indicated the presence of carbon atoms. The D band is also recognized as the disorder-induced band, being linked to structural defects or disorders present in the carbon material. The I_D_/I_G_ intensity ratio for Cr_2_AlC, Cr_3_AlC_2_, and Cr_4_AlC_3_ 0.86 suggests the presence of disorder and graphitization in the carbon structure (Patel et al., 2023).

### 3.2 Biomedical applications of Cr_2_AlC MAX phase, Cr_3_AlC_2_, and Cr_4_AlC_3_

**FIGURE 8:**
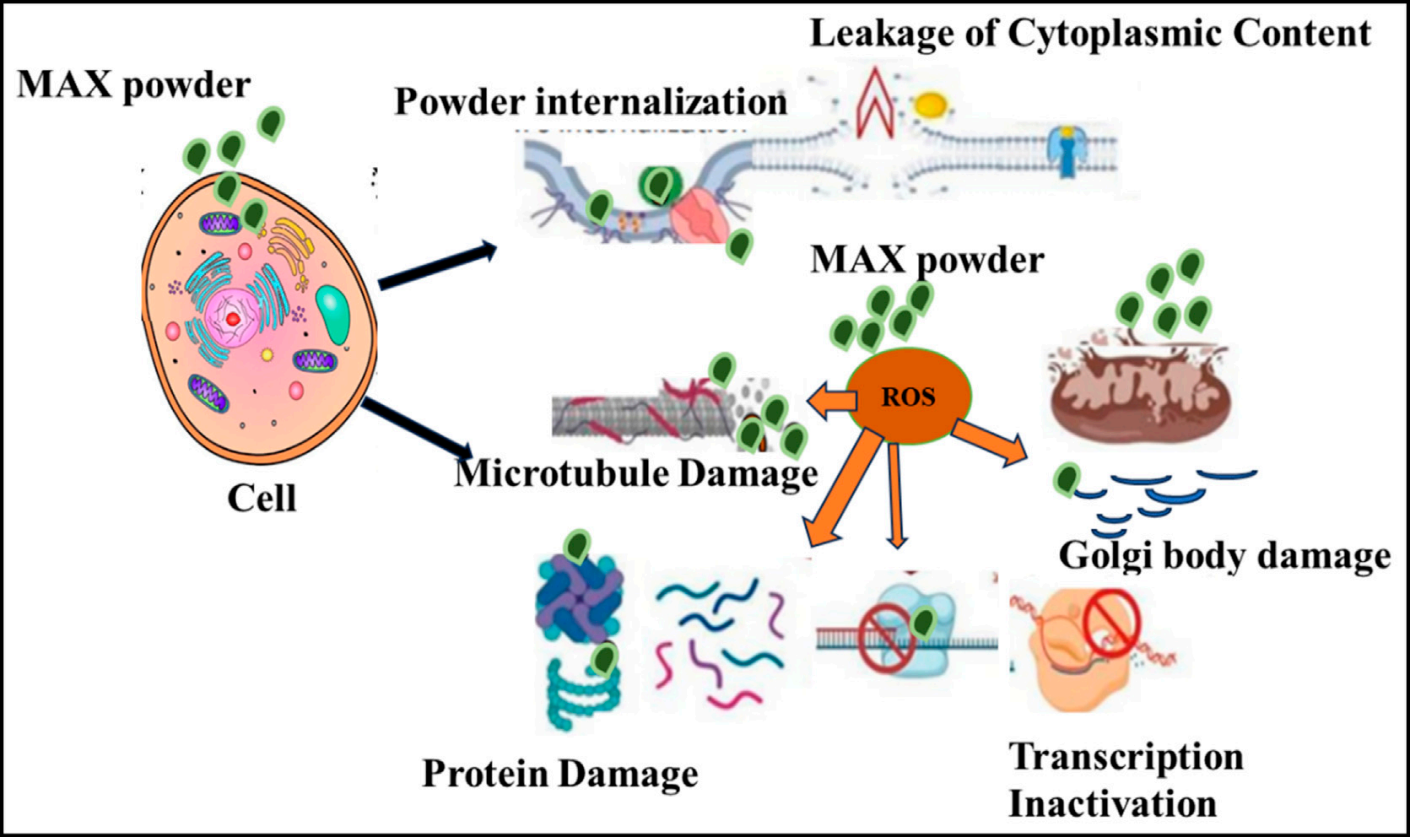
Schematic view of biomedical activity mechanism (antibacterial, antifungal, and anticancer) Activity.

The Cr_2_AlC MAX phase and prepared ternary compounds demonstrate a suppressive effect on the growth of microorganisms. It enhances the production of reactive oxygen species (ROS), which in turn disrupts the integrity of cell walls and membrane structures. This disruption impairs vital cellular functions such as DNA and RNA synthesis and increases the susceptibility of microorganisms to ROS. The approach for evaluating biomedical activity (such as antibacterial, antifungal, and anticancer effects) remains consistent remains consistent and its schematic view is shown in Figure 8.

**FIGURE 9:**
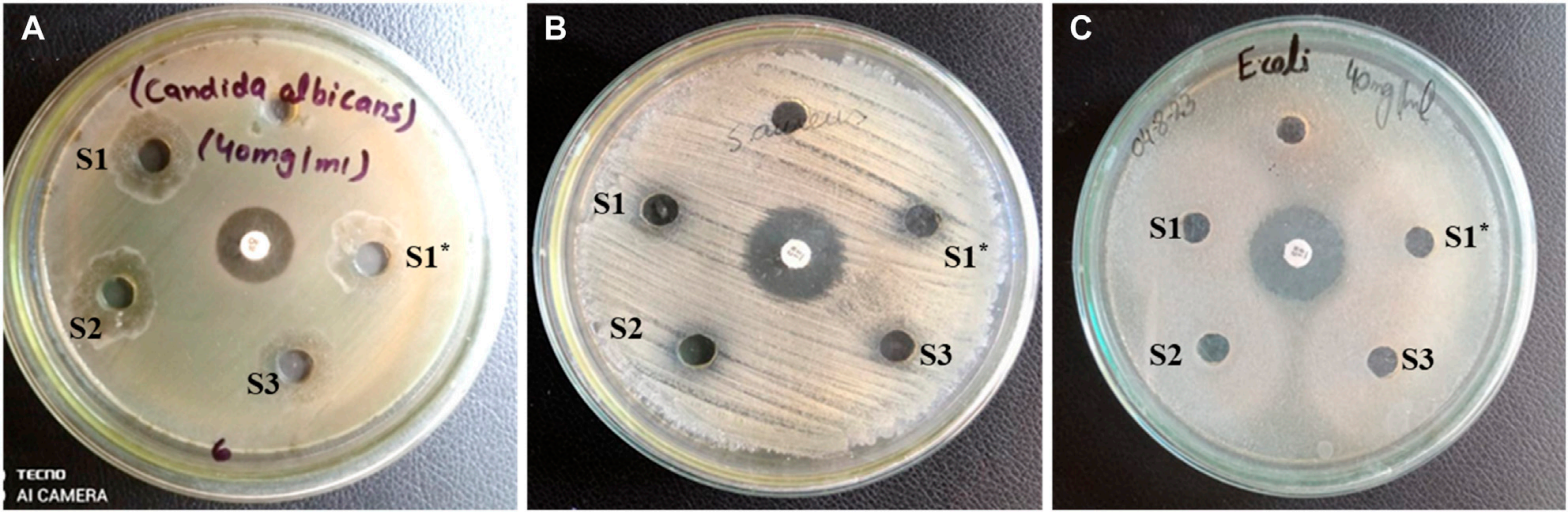
**(A)** Antifungal activity of S1, S1*(Cr_2_AlC MAX phase), S2 (Cr_3_AlC_2_), and S3 (Cr_4_AlC_3_) for *Candida albicans* **(B)** for S. aureus, and **(C)** for *E. coli*.

The antimicrobial activity of the samples synthesized via the sol-gel method was examined. It was found that these particles exhibit potent antimicrobial activity at a concentration of 40 mg/mL. In the context of this research article, we can delve into the biomedical applications of the synthesized Cr_2_AlC, Cr_3_AlC_2_, and Cr_4_AlC_3_ particularly focusing on its differential efficacy against *Candida albicans* and bacteria like *E. coli* and S. aureus (Chacko et al., 2024). Emphasize its noteworthy antifungal activity, demonstrated by an inhibition zone of 16 mm, 17 mm, and 15 mm (positive control = 15 mm) against *Candida albicans* for Cr_2_AlC, Cr_3_AlC_2_, and Cr_4_AlC_3_ respectively, underscoring its potential as an antifungal agent as shown in Figure 9 and Table 1. In contrast, it discussed its lack of effectiveness against both Gram-negative (*E. coli*) and Gram-positive (S.aureus) bacteria, exploring potential reasons for this selective antimicrobial activity. Consider factors such as the distinct interaction mechanisms of the Cr_2_AlC, Cr_3_AlC_2_, and Cr_4_AlC_3_ with fungal cells versus bacterial cells, or variations in cell wall structures and metabolic pathways between fungi and bacteria (Berardo et al., 2024).

**TABLE 1:** Antifungal activity of Cr_2_AlC, Cr_3_AlC_2_, and Cr_4_AlC_3_ for *Candida albicans*.

Sample	*Candida* *aAlbicans* dose (40 mg/mL) (Inhibition zones 15 mm)	*E. coli* Dose (40 mg/mL) (Inhibition zones 23 mm)	*S. aureus* Dose (40 mg/mL) (Inhibition zones 18 mm)
	Size (mm)	Size (mm)	Size (mm)
S1	16	—	6
S2	17	—	5
S3	15	—	7

To provide a comprehensive perspective on the biomedical potential of Cr_2_AlC, Cr_3_AlC_2_, and Cr_4_AlC_3_ materials, it is imperative to acknowledge their limitations and advocate for further research to enhance understanding and improve antibacterial properties. The antifungal properties of the Cr_2_AlC, Cr_3_AlC_2_, and Cr_4_AlC_3_ against *Candida albicans* can be ascribed to various inherent characteristics of nanomaterials like Cr_2_AlC, Cr_3_AlC_2_, and Cr_4_AlC_3_. Nanocomposites possess distinct physical properties, including a large specific surface area and unique interaction mechanisms with fungal cells, enabling effective targeting and inhibition of fungal growth. Conversely, the lack of efficacy against bacteria such as *E. coli* and *S. aureus* may be attributed to disparities in cell wall structures and metabolic pathways between fungi and bacteria (El-Zahed et al., 2024). Bacteria typically exhibit more complex and robust cell wall structures, potentially rendering them less susceptible to the mechanisms employed by Cr_2_AlC, Cr_3_AlC_2_, and Cr_4_AlC_3_ nanomaterials. Moreover, the interaction mechanisms of these nanomaterials may be more potent against fungi’s cell structures and reproductive mechanisms than bacteria. This specificity in antimicrobial activity underscores the significance of comprehending the intricate interactions between nanomaterials and various microorganisms for the advancement of targeted antimicrobial therapies (Warsi et al., 2022; Krasian et al., 2024).

### 3.3 Anti-cancerous activity

**FIGURE 10:**
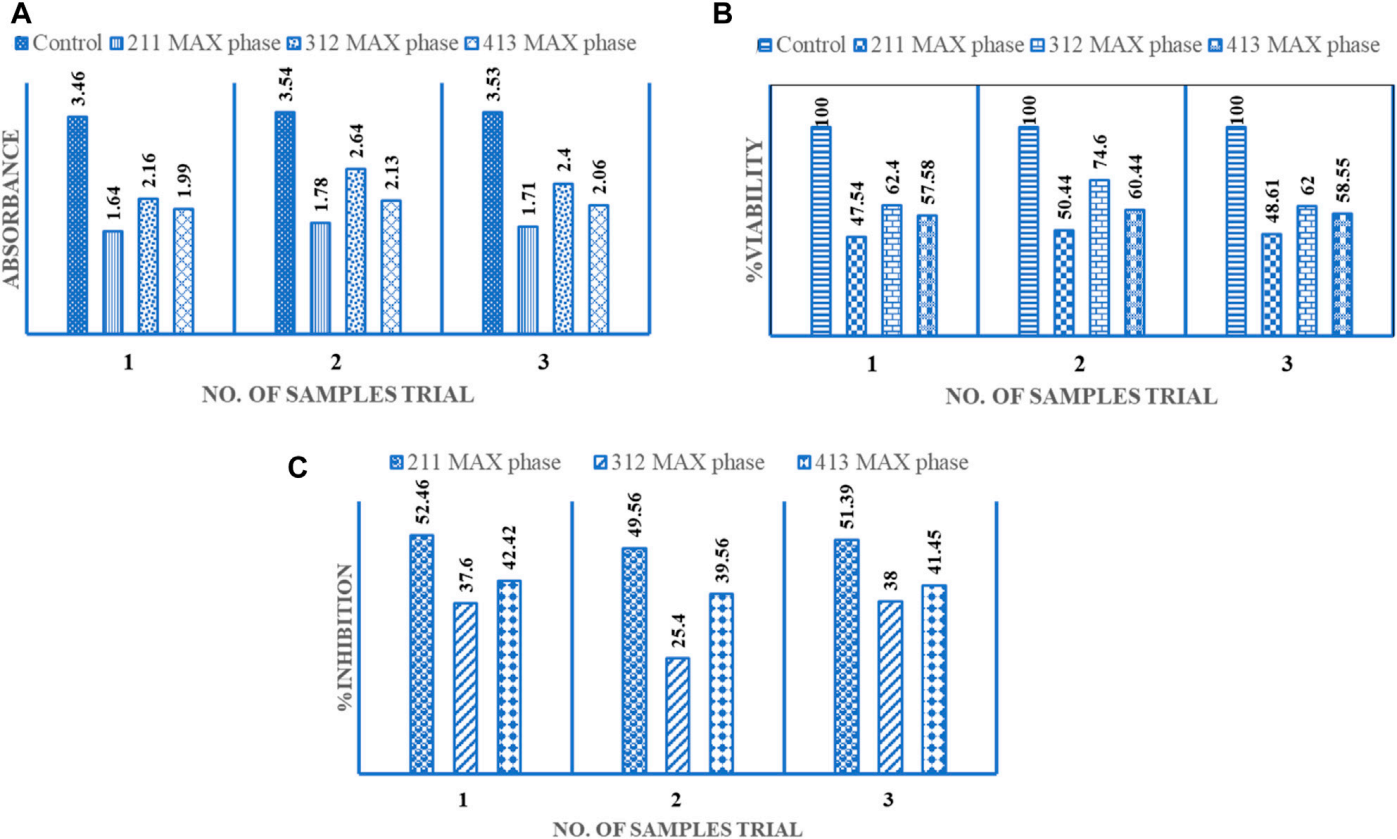
Anti-cancerous activity of Cr_2_AlC, Cr_3_AlC_2_, and Cr_4_AlC_3_ for HepG2 cell line **(A)** Absorbance, **(B)** %Viability and **(C)** % Inhibition.

The established medical use of synthesized Cr_2_AlC, Cr_3_AlC_2_, and Cr_4_AlC_3_ extends to treating various types of cancer cells (Khaled et al., 2024). The prepared samples of Cr_2_AlC, Cr_3_AlC_2_, and Cr_4_AlC_3_ were evaluated for their anti-cancer activity against liver cancer HepG2 cell line was conducted. The cells underwent treatment with a 200 μg/mL dosage for 48 h. In this investigation, the cytotoxic impact of Cr_2_AlC, Cr_3_AlC_2_, and Cr_4_AlC_3_ was evaluated on HepG-2 (human hepatocellular cancer cells), uncovering pronounced cytotoxicity against the HepG-2 cells. Particularly, at a concentration of 200 μg/mL, that composite exhibited noteworthy anti-cancer activity by markedly inhibiting the growth of hepatocellular carcinoma and reducing cellular viability in HepG-2 cells, as illustrated in Figure 10 (Al-Thubaiti et al., 2022; Kumar et al., 2024). Three distinct plots illustrate data for absorbance, Viability%, and % inhibition in the graphical depiction. The minimum absorbance value was observed as 1.64 (mean value 1.71, S. D 0.05), 2.4(mean value 2.4, S. D 0.19), and 1.99 (mean 2.06, S. D 0.05) for Cr_2_AlC, Cr_3_AlC_2_, and Cr_4_AlC_3_ respectively, as showed in Figure 10A and the maximum viability value was recorded as 50.4% (mean value 48.86, S. D 1.46), 62.4 (mean value 66.3, S. D 5.84) and 60.4 (mean value 58.8, S. D 1.18) as indicated in Figure 10B for Cr_2_AlC, Cr_3_AlC_2_, and Cr_4_AlC_3_ respectively. Figure 10C represented the maximum inhibition value observed as 52.46% (mean value 51.13, S. D 1.19), 38 (mean value 33.6, S. D 5.84), and 42.4 (mean value 41.1, S. D 1.18)for Cr_2_AlC, Cr_3_AlC_2_, and Cr_4_AlC_3_ respectively (Sedky et al., 2024).

## 4 Conclusion

In the initial attempt, a cost-effective sol-gel approach was utilized to synthesize Cr_2_AlC, Cr_3_AlC_2_, and Cr_4_AlC_3_ MAX phases. This novel synthesis method successfully produced these compounds through a sol-gel wet chemistry approach. Various characterization techniques were employed to confirm the formation of the MAX phases. The synthesized powders exhibited hexagonal structures, indicating high purity and stability. One notable advantage of this wet chemical technique was its enhanced handling of the precursor mixture, resulting in the formation of lamellar sheet structures for Cr_2_AlC, Cr_3_AlC_2_, and Cr_4_AlC_3_. Analysis of X-ray diffraction data confirmed the presence of highly crystalline Cr_2_AlC, Cr_3_AlC_2_, and Cr_4_AlC_3_, along with minor quantities of additional phases such as Cr_2_O_3_ and Cr_3_C_2_. Modifying the precursor mixture by increasing the excess of citric acid showed promise in reducing the level of oxide content in the final products. Furthermore, the synthesized materials exhibited notable antifungal efficacy against *Candida albicans*. Their anti-cancer activity was evaluated against the HepG2 cell line, with minimum absorbance values observed as follows: 1.64 (mean value 1.71, S.D 0.05) for Cr_2_AlC, 2.4 (mean value 2.4, S.D 0.19) for Cr_3_AlC_2_, and 1.99 (mean value 2.06, S.D 0.05) for Cr_4_AlC_3_. Maximum viability values were recorded as 50.4% (mean value 48.86, S.D 1.46) for Cr_2_AlC, 62.4% (mean value 66.3, S.D 5.84) for Cr_3_AlC_2_, and 60.4% (mean value 58.8, S.D 1.18) for Cr_4_AlC_3_. Additionally, maximum inhibition values were observed as 52.46% (mean value 51.13, S.D 1.19) for Cr_2_AlC, 38% (mean value 33.6, S.D 5.84) for Cr_3_AlC_2_, and 42.4% (mean value 41.1, S.D 1.18) for Cr_4_AlC_3_.

## Data Availability

The raw data supporting the conclusion of this article will be available upon request.

## References

[B1] Abdelkader A. M. (2016). Molten salts electrochemical synthesis of Cr_2_AlC. J. Eur. Ceram. Soc. 36 (1), 33–42. 10.1016/j.jeurceramsoc.2015.09.003

[B2] Al-Thubaiti E. H. El-Megharbel S. M. Albogami B. Hamza R. Z. (2022). Synthesis, spectroscopic, chemical characterizations, anticancer capacities against HepG-2, antibacterial and antioxidant activities of cefotaxime metal complexes with Ca (II), Cr (III), Zn (II), Cu (II) and Se (IV). Antibiotics 11 (7), 967. 10.3390/antibiotics11070967 35884222 PMC9311782

[B3] Bakht J. Islam A. Shafi M. (2011). Antimicrobial potential of Eclipta alba by well diffusion method. Pak. J. Bot. 43, 161–166.

[B4] Bentzel G. W. Naguib M. Lane N. J. Vogel S. C. Presser V. Dubois S. (2016). High‐temperature neutron diffraction, Raman spectroscopy, and first‐principles calculations of Ti_3_SnC_2_ and Ti_2_SnC. Raman Spectrosc. first‐principles Calc. Ti_3_SnC_2_ Ti_2_SnC J. Am. Ceram. Soc. 99 (7), 2233–2242. 10.1111/jace.14210

[B5] Berardo M. E. V. Mendieta J. R. Villamonte M. D. Colman S. L. a D. N. (2024). Antifungal and antibacterial activities of Cannabis sativa L. resins. J. Ethnopharmacol. 318, 116839. 10.1016/j.jep.2023.116839 37400009

[B6] Bortolozo A. Serrano G. Serquis A. Rodrigues D. Jr Dos Santos C. Fisk Z. (2010). Superconductivity at 7.3 K in Ti_2_InN. Solid State Commun. 150 (29-30), 1364–1366. 10.1016/j.ssc.2010.04.036

[B7] Bortolozo A. D. Sant’Anna O. H. Santos C. A. M. d. Machado A. J. S. (2007). Superconductivity in the hexagonal-layered nanolaminates Ti_2_InC compound. Solid State Commun. 144 (10-11), 419–421. 10.1016/j.ssc.2007.09.028

[B8] Chacko S. K. Balakrishnan R. Kalarikkal N. Thomas N. G. (2024). Ternary fiber mats of PVDF-HFP/cellulose/LiFe5O8 nanoparticles with enhanced electric, magnetoelectric, and antibacterial properties: a promising approach for magnetic and electric field-responsive antibacterial coatings. ACS Appl. Polym. Mater. 6 (2), 1429–1438. 10.1021/acsapm.3c02588

[B9] Crisan O. Crisan A. D. (2018). Incipient low-temperature formation of MAX phase in Cr–Al–C films. J. Adv. Ceram. 7, 143–151. 10.1007/s40145-018-0265-5

[B10] Duan X. Shen L. Jia D. Zhou Y. Zwaag S. v. d. Sloof W. G. (2015). Synthesis of high-purity, isotropic or textured Cr_2_AlC bulk ceramics by spark plasma sintering of pressure-less sintered powders. J. Eur. Ceram. Soc. 35 (5), 1393–1400. 10.1016/j.jeurceramsoc.2014.11.008

[B11] El-Zahed M. M. Diab M. A. El-Sonbati A. Z. Saad M. H. Eldesoky A. M. El-Bindary M. A. (2024). Synthesis, spectroscopic characterization studies of chelating complexes and their applications as antimicrobial agents, DNA binding, molecular docking, and electrochemical studies. Appl. Organomet. Chem. 38 (1), e7290. 10.1002/aoc.7290

[B12] Galvin T. Hyatt N. C. Rainforth W. M. Reaney I. M. Shepherd D. (2018). Molten salt synthesis of MAX phases in the Ti-Al-C system. J. Eur. Ceram. Soc. 38 (14), 4585–4589. 10.1016/j.jeurceramsoc.2018.06.034

[B13] Gao L. Han T. Guo Z. Zhang X. Pan D. Zhou S. (2020). Preparation and performance of MAX phase Ti_3_AlC_2_ by *in-situ* reaction of Ti-Al-C system. Adv. Powder Technol. 31 (8), 3533–3539. 10.1016/j.apt.2020.06.042

[B14] Gonzalez-Julian J. (2021). Processing of MAX phases: from synthesis to applications. J. Am. Ceram. Soc. 104 (2), 659–690. 10.1111/jace.17544

[B15] Gorshkov V. A. Miloserdov P. A. Luginina M. A. Sachkova N. V. Belikova A. F. (2017). High-temperature synthesis of a cast material with a maximum content of the MAX phase Cr_2_AlC. Inorg. Mater. 53, 271–277. 10.1134/s0020168517030062

[B16] Gorshkov V. A. Miloserdov P. A. Sachkova N. V. Luginina M. A. Yukhvid V. I. (2018). SHS metallurgy of Cr_2_AlC MAX phase-based cast materials. Russ. J. Non-Ferrous Metals 59, 570–575. 10.3103/s106782121805005x

[B17] Guan C. (2016). Synthesis of fine and high purity Cr_2_AlC powders by novel method. Adv. Appl. Ceram. 115 (8), 505–508. 10.1179/1743676115y.0000000026

[B18] Hauert R. Patscheider J. Tobler M. Zehringer R. (1993). XPS investigation of the aC: H/Al interface. Surf. Sci. 292 (1-2), A605–A129. 10.1016/0167-2584(93)90851-9

[B19] Hench L. L. West J. K. (1990). The sol-gel process. Chem. Rev. 90 (1), 33–72. 10.1021/cr00099a003

[B20] Kaya G. Koc E. O. Özdemir S. Yalçın M. S. Ocakoglu K. Dizge N. (2024). The syntheses of chromium aluminum carbide (Cr_2_AlC) MAX phase and Cr_2_CT_x_ MXene and investigation of their antimicrobial properties. Appl. Biochem. Biotechnol., 1–15. 10.1007/s12010-024-04910-w 38538871

[B21] Khaled A. M. Othman M. S. Obeidat S. T. Aleid G. M. Aboelnaga S. M. Fehaid A. (2024). Green-synthesized silver and selenium nanoparticles using berberine: a comparative assessment of *in vitro* anticancer potential on human hepatocellular carcinoma cell line (HepG2). Cells 13 (3), 287. 10.3390/cells13030287 38334679 PMC10854975

[B22] Krasian T. Punyodom W. Molloy R. Topham P. D. Tighe B. J. Mahomed A. (2024). Low cytotoxicity, antibacterial property, and curcumin delivery performance of toughness-enhanced electrospun composite membranes based on poly (lactic acid) and MAX phase (Ti_3_AlC_2_). International Journal of Biological Macromolecules, 129967. Int. J. Biol. Macromol. 262, 129967. 10.1016/j.ijbiomac.2024.129967 38316324

[B23] Kumar D. Pal R. R. Das N. Roy P. Saraf S. A. Bayram S. (2024). Synthesis of flaxseed gum/melanin-based scaffold: a novel approach for nano-encapsulation of doxorubicin with enhanced anticancer activity. Int. J. Biol. Macromol. 256, 127964. 10.1016/j.ijbiomac.2023.127964 37951423

[B24] Lei X. Lin N. (2022). Structure and synthesis of MAX phase materials: a brief review. Crit. Rev. Solid State Mater. Sci. 47 (5), 736–771. 10.1080/10408436.2021.1966384

[B25] Li Y. Zhao G. Qian Y. Xu J. Li M. (2018). Deposition of phase-pure Cr_2_AlC coating by DC magnetron sputtering and post annealing using Cr-Al-C targets with controlled elemental composition but different phase compositions. J. Mater. Sci. Technol. 34 (3), 466–471. 10.1016/j.jmst.2017.01.029

[B26] Li Z. Wang Z. Ma G. Chen R. Yang W. Wang K. (2024). High-performance Cr_2_AlC MAX phase coatings for ATF application: interface design and oxidation mechanism. Corros. Commun. 13, 27–36. 10.1016/j.corcom.2023.10.001

[B27] Lin Z. Zhou Y. Li M. Wang J. (2022). *In-situ* hot pressing/solid-liquid reaction synthesis of bulk Cr_2_AlC. Int. J. Mater. Res. 96 (3), 291–296. 10.3139/146.101033

[B28] Liu J. Zuo X. Wang Z. Wang L. Wu X. Ke P. (2018). Fabrication and mechanical properties of high purity of Cr_2_AlC coatings by adjustable Al contents. J. Alloys Compd. 753, 11–17. 10.1016/j.jallcom.2018.04.100

[B29] Liu P. Hu M. Hu L. Yin M. Wu H. Hu M. (2020). Fabrication of Cr_2_AlC powder by molten salt electrolysis at 850° C with good oxidation resistance. J. Alloys Compd. 826, 154003. 10.1016/j.jallcom.2020.154003

[B30] Liu Z. Xu J. Xi X. Luo W. Zhou J. (2023). Molten salt dynamic sealing synthesis of MAX phases (Ti_3_AlC_2_, Ti_3_SiC_2_ et al.) powder in air. Ceram. Int. 49 (1), 168–178. 10.1016/j.ceramint.2022.08.325

[B31] Manoun B. Gulve R. P. Saxena S. K. Gupta S. Barsoum M. W. Zha C. S. (2006). Compression behavior of M_2_AlC (M= Ti, V, Cr, Nb, and Ta) phases to above 50 GPa. Phys. Rev. B 73 (2), 024110. 10.1103/physrevb.73.024110

[B32] Mansouri B. Rafiei M. Ebrahimzadeh I. Naeimi F. Barekat M. (2023). The effect of milling time and heat treatment on the synthesis of the Cr_2_AlC MAX phase. Can. Metall. Q., 1–11. 10.1080/00084433.2023.2251210

[B33] Monireh A. Alireza K. Samira A.-O. Behrouz V. Yasin O. Yeojoon Y. (2023a). Catalytic activation of hydrogen peroxide by Cr_2_AlC MAX phase under ultrasound waves for a treatment of water contaminated with organic pollutants. Ultrason. Sonochemistry 93, 106294. 10.1016/j.ultsonch.2023.106294 PMC9852641 36640461

[B34] Monireh A. Alireza K. Samira A.-O. Behrouz V. Yasin O. Yeojoon Y. (2023b). Catalytic activation of hydrogen peroxide by Cr_2_AlC MAX phase under ultrasound waves for a treatment of water contaminated with organic pollutants. Ultrason. Sonochemistry 93, 106294. 10.1016/j.ultsonch.2023.106294 PMC9852641 36640461

[B35] Patel P. C. Mishra P. K. Kandpal H. C. (2023). Study of MAX phase based Schottky interfacial structure: the case of electron-beam deposited epitaxial Cr_2_AlC film on p–Si (100). J. Mater. Sci. 58 (9), 4041–4053. 10.1007/s10853-023-08286-w

[B36] Poulou A. Mellan T. A. Finnis M. W. (2021). Stability of Zr-Al-C and Ti-Al-C MAX phases: a theoretical study. A Theor. study. Phys. Rev. Mater. 5 (3), 033608. 10.1103/physrevmaterials.5.033608

[B37] Presser V. Naguib M. Chaput L. Togo A. Hug G. Barsoum M. W. (2012). First‐order Raman scattering of the MAX phases: Ti_2_AlN, Ti_2_AlC_0. 5_N_0. 5_, Ti_2_AlC,(Ti_0. 5_V_0. 5_) _2_AlC, V_2_AlC, Ti_3_AlC_2_, and Ti_3_GeC2 43(1), 168-172. J. Raman Spectrosc. 43 (1), 168–172. 10.1002/jrs.3036

[B38] Rajkumar Y. Rahul B. M. Akash P. A. Panigrahi B. B. (2017). Nonisothermal sintering of Cr_2_AlC powder. Int. J. Appl. Ceram. Technol. 14 (1), 63–67. 10.1111/ijac.12617

[B39] Rampai H. Tokoloho L. (2011). Synthesis of Ti₂AlC, Ti₃AlC₂ and Ti₃SiC₂ MAX phase ceramics; and their composites with c-BN.

[B40] Reghunath B. S. Davis D. Devi K. R. S. (2021). Synthesis and characterization of Cr_2_AlC MAX phase for photocatalytic applications. Chemosphere 283, 131281. 10.1016/j.chemosphere.2021.131281 34467941

[B41] Rueß H. Werner J. Unutulmazsoy Y. Gerlach J. W. Chen X. Stelzer B. (2021). Effect of target peak power density on the phase formation, microstructure evolution, and mechanical properties of Cr_2_AlC MAX-phase coatings. J. Eur. Ceram. Soc. 41 (3), 1841–1847. 10.1016/j.jeurceramsoc.2020.10.072

[B42] Sarkar S. Banerjee P. Raychaudhury M. D. (2024). The Elusive member of the Ti-Al-C MAX family-Ti_4_AlC_3_ . arXiv preprint arXiv:2402.10621.

[B43] Sedky N. K. Fawzy I. M. Hassan A. Mahdy N. K. Attia R. T. Shamma S. N. (2024). Innovative microwave-assisted biosynthesis of copper oxide nanoparticles loaded with platinum (ii) based complex for halting colon cancer: cellular, molecular, and computational investigations. RSC Adv. 14 (6), 4005–4024. 10.1039/d3ra08779d 38288146 PMC10823359

[B44] Shalini Reghunath B. Davis D. Sunaja Devi K. R. (2021). Synthesis and characterization of Cr2AlC MAX phase for photocatalytic applications. Chemosphere 283, 131281. 10.1016/j.chemosphere.2021.131281 34467941

[B45] Shamsipoor A. Farvizi M. Razavi M. Keyvani A. Mousavi B. Pan W. (2021). Hot corrosion behavior of Cr_2_AlC MAX phase and CoNiCrAlY compounds at 950° C in presence of Na_2_SO_4_+ V_2_O_5_ molten salts. Ceram. Int. 47 (2), 2347–2357. 10.1016/j.ceramint.2020.09.077

[B46] Sharma P. Kainth S. Singh K. Mahajan R. L. Pandey O. P. (2023). Investigating non-isothermal oxidation kinetics of a non-stoichiometrically synthesized Ti_3_AlC_2_ MAX phase. J. Alloys Compd. 959, 170488. 10.1016/j.jallcom.2023.170488

[B47] Sharma P. Pandey O. P. (2019). Non-isothermal oxidation kinetics of nano-laminated Cr_2_AlC MAX phase. J. Alloys Compd. 773, 872–882. 10.1016/j.jallcom.2018.09.326

[B48] Sharmin S. Rahaman M. M. Sarkar C. Atolani O. Islam M. T. Adeyemi O. S. (2021). Nanoparticles as antimicrobial and antiviral agents: a literature-based perspective study. Heliyon 7 (3), e06456. 10.1016/j.heliyon.2021.e06456 33763612 PMC7973307

[B49] Shtansky D. V. Kiryukhantsev-Korneev P. V. Sheveyko A. N. Mavrin B. N. Rojas C. Fernandez A. (2009). Comparative investigation of TiAlC (N), TiCrAlC (N), and CrAlC (N) coatings deposited by sputtering of МАX-phase Ti_2− X_Cr_X_AlC targets. Surf. Coatings Technol. 203 (23), 3595–3609. 10.1016/j.surfcoat.2009.05.036

[B50] Siebert J. P. Bischoff L. Lepple M. Zintler A. Molina-Luna L. Wiedwald U. (2019). Sol–gel-based synthesis and enhanced processability of MAX phase Cr_2_GaC. J. Mater. Chem. C 7 (20), 6034–6040. 10.1039/c9tc01416k

[B51] Soundiraraju B. Raghavan R. George B. K. (2020). Chromium carbide nanosheets prepared by selective etching of aluminum from Cr_2_AlC for hydrazine detection. ACS Appl. Nano Mater. 3 (11), 11007–11016. 10.1021/acsanm.0c02230

[B52] Spanier J. E. Gupta S. Amer M. S. Barsoum M. W. (2005). Vibrational behavior of the Mn phases from first-order Raman scattering Raman scattering (M= Ti, V, Cr,= Ti, V, Cr, A= Si, X= C, N). Phys. Rev. B Phys. Rev. B 71, 012103.

[B53] Sun Z. M. (2011). Progress in research and development on MAX phases: a family of layered ternary compounds. Int. Mater. Rev. 56 (3), 143–166. 10.1179/1743280410y.0000000001

[B54] Ta Q. T. H. Tran N. M. Noh J.-S. (2021). Pressureless manufacturing of Cr_2_AlC compound and the temperature effect. Mater. Manuf. Process. 36 (2), 200–208. 10.1080/10426914.2020.1819547

[B55] Tan L. Guan C. Tian Y. Dang P. Wang S. Li J. (2019). Synthesis and tribological properties of ultrafine Cr_2_AlC MAX phase. Ceram. Soc. Jpn. 127 (10), 754–760. 10.2109/jcersj2.18184

[B56] Tian W. Sun Z. Du Y. Hashimoto H. (2008). Synthesis reactions of Cr_2_AlC from Cr–Al_4_C_3_–C by pulse discharge sintering. Mater. Lett. 62 (23), 3852–3855. 10.1016/j.matlet.2008.05.001

[B57] Tunes M. A. Https M. I. Kainz C. Pogatscher S. Vishnyakov V. M. (2021). Deviating from the pure MAX phase concept: radiation-tolerant nanostructured dual-phase Cr_2_AlC. Sci. Adv. 7 (13), eabf6771. 10.1126/sciadv.abf6771 33762345 PMC7990341

[B58] Tzenov N. V. Barsoum M. W. (2000). Synthesis and characterization of Ti_3_AlC_2_ . J. Am. Ceram. Soc. 83 (4), 825–832. 10.1111/j.1151-2916.2000.tb01281.x

[B59] Vishnyakov V. Crisan O. Dobrosz P. Colligon J. S. (2014). Ion sputter-deposition and in-air crystallisation of Cr_2_AlC films. Vacuum 100, 61–65. 10.1016/j.vacuum.2013.07.045

[B60] Warsi A.-Z. Aziz F. Zulfiqar S. Haider S. Shakir I. Agboola P. O. (2022). Synthesis, characterization, photocatalysis, and antibacterial study of WO_3_, MXene and WO_3_/MXene nanocomposite. Nanomaterials 12 (4), 713. 10.3390/nano12040713 35215041 PMC8877483

[B61] Wei G.-L. Li D.-Q. Zhuo M.-N. Liao Y.-S. Xie Z.-Y. Guo T.-L. (2015). Organophosphorus flame retardants and plasticizers: sources, occurrence, toxicity and human exposure. Environ. Pollut. 196, 29–46. 10.1016/j.envpol.2014.09.012 25290907

[B62] Yan M. Duan X. Zhang Z. Liao X. Zhang X. Qiu B. (2017). Nanometre-scale 3D defects in Cr_2_AlC thin films. Sci. Rep. 7 (1), 984. 10.1038/s41598-017-01196-3 28428564 PMC5430507

[B63] Yan M. Duan X. Zhang Z. Liao X. Zhang X. Qiu B. (2019). Effect of ball milling treatment on the microstructures and properties of Cr_2_AlC powders and hot pressed bulk ceramics. J. Eur. Ceram. Soc. 39 (16), 5140–5148. 10.1016/j.jeurceramsoc.2019.07.052

[B64] Younas M. Rizwan M. Zubair M. Inam A. Ali S. (2021). Biological synthesis, characterization of three metal-based nanoparticles and their anticancer activities against hepatocellular carcinoma HepG2 cells. Ecotoxicol. Environ. Saf. 223, 112575. 10.1016/j.ecoenv.2021.112575 34352575

[B65] Zamulaeva E. I. Levashov E. A. Skryleva E. A. Sviridova T. A. Kiryukhantsev-Korneev P. V. (2016). Conditions for formation of MAX phase Cr_2_AlC in electrospark coatings deposited onto titanium alloy. Surf. Coatings Technol. 298, 15–23. 10.1016/j.surfcoat.2016.04.058

[B66] Zamulaeva E. I. Levashov E. A. Sviridova T. A. Shvyndina N. V. Petrzhik M. I. (2013). Pulsed electrospark deposition of MAX phase Cr_2_AlC based coatings on titanium alloy. Surf. Coatings Technol. 235, 454–460. 10.1016/j.surfcoat.2013.08.002

[B67] Zhang p. Yan S. Li C. Ding Y. He J. Yin F. (2019). Synthesis and characterization of MAX phase Cr_2_AlC based composite coatings by plasma spraying and post annealing. J. Eur. Ceram. Soc. 39 (16), 5132–5139. 10.1016/j.jeurceramsoc.2019.08.039

[B68] Zhang Y. Wen J. Zhang L. Lu H. Guo Y. Ma X. (2021). High antioxidant lamellar structure Cr_2_AlC: dielectric and microwave absorption properties in X band. J. Alloys Compd. 860, 157896. 10.1016/j.jallcom.2020.157896

